# Cascading effects of N input on tritrophic (plant–aphid–parasitoid) interactions

**DOI:** 10.1002/ece3.2404

**Published:** 2016-10-11

**Authors:** Quentin Chesnais, Aude Couty, Manuella Catterou, Arnaud Ameline

**Affiliations:** ^1^ FRE CNRS 3498 EDYSAN (Écologie et Dynamique des Systèmes Anthropisés) Université de Picardie Jules Verne Amiens Cedex France

**Keywords:** Brassicaceae, *Brevicoryne brassicae*, *Camelina sativa*, *Diaeretiella rapae*, *Myzus persicae*, nitrogen fertilization, performances, tritrophic interactions hypothesis

## Abstract

Because N is frequently the most limiting mineral macronutrient for plants in terrestrial ecosystems, modulating N input may have ecological consequences through trophic levels. Thus, in agro‐ecosystems, the success of natural enemies may depend not only from their herbivorous hosts but also from the host plant whose qualities may be modulated by N input. We manipulated foliar N concentrations by providing to *Camelina sativa* plants three different nitrogen rates (control, optimal, and excessive). We examined how the altered host‐plant nutritional quality influenced the performances of two aphid species, the generalist green peach aphid, *Myzus persicae,* and the specialist cabbage aphid, *Brevicoryne brassicae*, and their common parasitoid *Diaeretiella rapae*. Both N inputs led to increased N concentrations in the plants but induced contrasted concentrations within aphid bodies depending on the species. Compared to the control, plant biomass increased when receiving the optimal N treatment but decreased under the excessive treatment. Performances of *M. persicae* improved under the optimal treatment compared to the control and excessive treatments whereas *B. brassicae* parameters declined following the excessive N treatment. In no‐choice trials, emergence rates of *D. rapae* developing in *M. persicae* were higher on both optimum and excessive N treatments, whereas they remained stable whatever the treatment when developing in *B. brassicae*. Size of emerging *D. rapae* females was positively affected by the treatment only when it developed in *M. persicae* on the excessive N treatment. This work showed that contrary to an optimal N treatment, when N was delivered in excess, plant suitability was reduced and consequently affected negatively aphid parameters. Surprisingly, these negative effects resulted in no or positive consequences on parasitoid parameters, suggesting a buffered effect at the third trophic level. Host N content, host suitability, and dietary specialization appear to be major factors explaining the functioning of our studied system.

## 1 Introduction

Trophic cascades, defined as mutual consumer–resource interactions that alter performances of trophic levels across more than one link in a food web (Knight, McCoy, Chase, McCoy, & Holt, 2005), represent not only indirect effects of a higher trophic level on nonadjacent lower trophic levels, that is top‐down cascade, but also effects of lower trophic levels that can transmit upward to nonadjacent higher trophic levels, that is bottom‐up cascade (Hunter & Price, 1992; Johnson, 2008).

Ecological stoichiometry studies these interactions in ecosystems by investigating how the balance of energy and elements affects and is affected by organisms (Moe et al., 2005; Sterner & Elser, 2002). Indeed, there are differences in the nitrogen (N) content among trophic levels indicating that consumer development would be limited by the amount of N in the resources (White, 1993). The amount of N is an important factor limiting survival and growth of several herbivores and predators (Denno & Fagan, 2003; Mattson, 1980). Therefore, N in host plants and its accumulation by herbivorous insects may influence the performance of natural enemies (Kagata & Ohgushi, 2006). One hypothesis for explaining trophic cascades is that a higher N level in host plants leads to an increase in the N content of herbivorous insects, which in turn leads to an increase in predator performance (Mayntz & Toft, 2001).

In terrestrial agro‐ecosystems, nitrogen (N) is an important macronutrient for plants that may vary and be patchy due to soil properties (Keddy, 2007). N availability strongly defines patterns of plant growth and allocation. Generally, in response to enhanced N, plant foliar N concentration, photosynthetic rate, growth rate, and aerial biomass production increase (Hermans, Hammond, White, & Verbruggen, 2006; Leith, Hicks, Fowler, & Woodin, 1999; Magill et al., 2000). However, excessive N input may induce symptoms indicating toxicity such as chlorosis of leaves, reduced growth, or yield depressions which are due to excessive ammonium (NH4+) in plants (Britto & Kronzucker, 2002). Because N is frequently the most limiting mineral macronutrient for plants in temperate terrestrial ecosystems (Vitousek & Howarth, 1991), modulating N input may have ecological consequences through the different trophic levels.

Generally, improved plant nutritional quality due to enhanced leaf N content (i.e., decreased C:N ratio) has been reported to induce better herbivore development (Awmack & Leather, 2002; Huberty & Denno, 2006; Stiling & Moon, 2005; Zehnder & Hunter, 2008) and higher herbivore population growth rate (Gratton & Denno, 2003). However, using an aphid model and by experimentally manipulating foliar N content, Zehnder and Hunter (2009) have shown that strong N foliar content due to excessive N input may limit herbivore population growth rates.

Within agro‐ecosystems, nitrogen can exert a variety of bottom‐up effects through the different trophic levels, from the plant to the natural enemies (predators/parasitoids). Development and life‐history traits of parasitoids directly depend upon their host quality and, indirectly, upon the quality of the host plant (Schädler, Brandl, & Kempel, 2010; Turlings & Benrey, 1998). Plant nitrogen inputs were reported to directly improve host nutritional quality for parasitoid development through an increase of N concentrations in host bodies (Winter & Rostás, 2010). Several studies have shown that N plant inputs generally enhance parasitism success (Aqueel et al., 2015; Ponti, Altieri, & Gutierrez, 2007), but to our knowledge, the impact of excessive N input on the third level has not been investigated yet. Nevertheless, N is known to cascade up to higher trophic levels and subsequently alter the abundance and/or performance of natural enemies, thus altering top‐down influences.

This work explores how different levels of N input (no input, optimum input, excessive input) impact host‐plant quality (biomass and C:N ratio) and subsequently may alter tritrophic interactions. As aphids have proven to be useful and interesting models for studying important questions in ecology and evolution (Huang & Qiao, 2014), two aphid species (*Myzus persicae* and *Brevicoryne brassicae*) differing in their dietary specialization and being both used as hosts by the aphid parasitoid wasp *Diaeretiella rapae* Mc'Intosh (Hymenoptera: Braconidae) were studied on the host plant *Camelina sativa* (Brassicales: Brassicaceae). Increasing attention has been paid to camelina, both because of its phyletic proximity to *Arabidopsis thaliana* (Beilstein, Al‐Shehbaz, Mathews, & Kellogg, 2008) and its agronomic characteristics (Bansal & Durrett, 2016). In terms of yield, camelina responded positively to nitrogen input up to 80–120 kgN/ha and negatively to higher N input (Solis, Vidal, Paulino, Johnson, & Berti, 2013). Camelina is generally reported to be tolerant and resistant to various pathogens and insects (Séguin‐Swartz et al., 2009), although it has recently been shown to be a potential host for some aphid pests (Chesnais, Verzeaux, Couty, Le Roux, & Ameline, 2015). Among those aphids, *B. brassicae* L. (Hemiptera: Aphididae) and *M. persicae* Sulzer (Hemiptera: Aphididae) are commonly studied on Brassicaceae crops because of their different dietary specialization. *Brevicoryne brassicae* is a Brassicaceae specialist (Costello & Altieri, 1995), responsible for severe damage on cabbage (Blackman & Eastop, 2000). *M. persicae* is a generalist aphid (Costello & Altieri, 1995) and a serious pest on a wide range of crops (Cole, 1997b).

In our study, different N fertilization rates (optimum vs. excessive) were used to modulate *C. sativa* nitrogen availability to determine whether altered leaf N content would cascade up through trophic levels and then would influence the interaction between the two aphid species and their common aphid parasitoid, *D. rapae*. We expected that insect performances would depend on their host (either the host plant for the aphid or the host aphid for the parasitoid) N concentration. The “tritrophic interactions” (TTI) hypothesis predicts that generalist herbivores should be more sensitive to variations in host‐plant quality than specialist herbivores, and thus that the subsequent effects on natural enemies should be more important when the generalist host/prey feeds on low‐quality plants (Mooney, Pratt, & Singer, 2012). We therefore expected that the variation in host‐plant quality would affect more the parasitoid *D. rapae* developing on *M. persicae* than when it developed on *B. brassicae*.

## 2 Materials and Methods

### 2.1 Insects

The *M. persicae* (Sulzer) colony was established from one parthenogenetic female collected in 1999 in a potato field near Loos‐en‐Gohelle (France). The *B. brassicae* (L.) colony, established from one parthenogenetic female, was provided in 2008 by INRA‐Le Rheu (Rennes, France). Aphids of both colonies were reared on rapeseed (*Brassica napus cv*. “Adriana”). Pots (90 × 90 × 70 mm) containing each three to four rapeseed plants were placed in ventilated plastic cages (240 × 110 × 360 mm) and maintained in a growth chamber under 20 ± 1°C, 60% ± 5% relative humidity (RH), and 16L:8D photoperiod at 2.5 klux. Aphid clones were used to minimize intraspecific variability and to ensure a certain uniformity of response.

*Diaeretiella rapae* (M'Intosh, 1855) parasitoids (Hymenoptera: Aphidiidae) were obtained from IFTECH (Les Ponts de Cé, France) as mummies and were reared on the complex *B. brassicae*–*B. napus*. Upon reception, mummies were transferred to plastic tubes (75 × 13 mm) closed with a cotton plug. Once emerged, parasitoids were sexed and mating was allowed by grouping three to four males with six to seven females in the same tube. They were fed ad libitum with a 1:1 honey/water (v/v) solution until used for the experiments. Parasitoids were maintained in a room at 20 ± 1°C, 60% ± 5% RH, and a 16L:8D photoperiod at 2.5 klux. Three‐day‐old standardized parasitoid females (mated, fed, and without oviposition experience) and males (mated and fed) were randomly selected for the experiments.

### 2.2 Plants and nitrogen treatments

**Figure 1:**
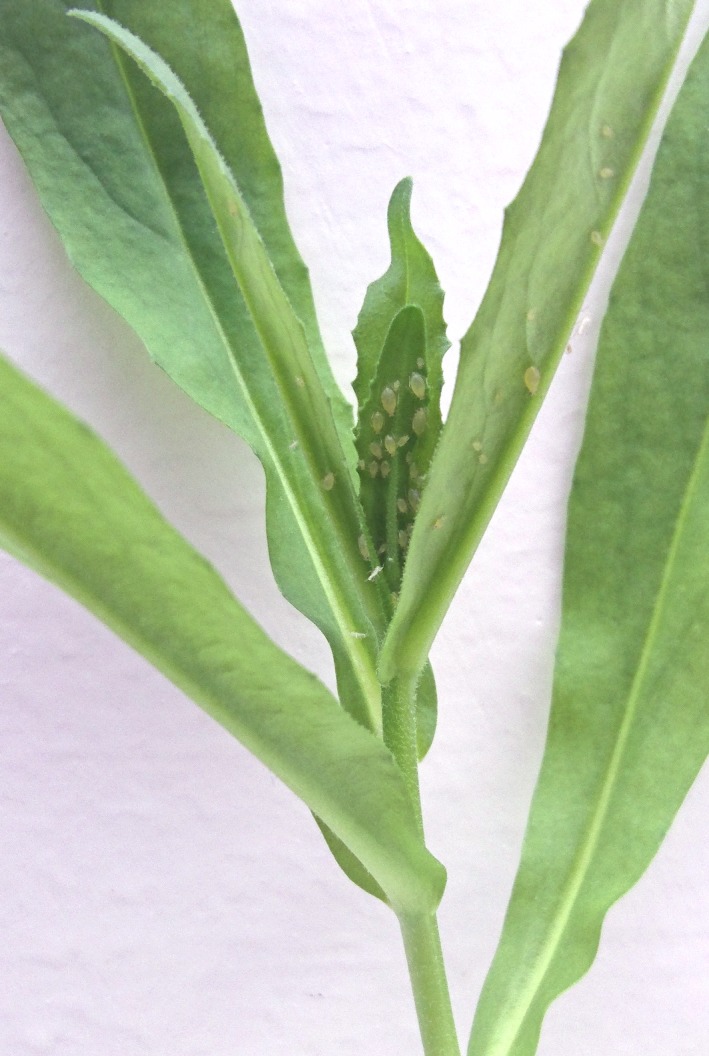
Green peach aphid, *Myzus persicae*, on *Camelina sativa* plant

Camelina seeds (*Camelina sativa cv*. “Celine”) were sown (4 seeds/pot) in plastic pots (9 × 9 × 10 cm) containing potting soil (NPK 18‐10‐20, 0.5 kg/m^3^, FLORAGARD) and maintained in a growth chamber under 20 ± 1°C, 60% ± 5% RH, and 16L:8D photoperiod at 2.5 klux (Fig. 1).

Plants were randomly assigned to one of the three following treatments: (1) no nitrogen input, consisting of the potting soil only (control referred to as “0N”); (2) potting soil supplemented with 0.065 gN/pot (i.e., 80 kgN/ha) of ammonium nitrate (referred to as “80N”), constituting the recommended input for camelina (Solis et al., 2013); or (3) potting soil supplemented with 0.195 gN/pot (i.e., 240 kgN/ha) of ammonium nitrate (referred to as “240N”). Application of the nitrogenous solutions was carried out over the entire soil surface of each pot. A first half‐dose, diluted in 100 ml of water, was applied at the sowing date. A second half‐dose was applied 1 week later. Four‐week‐old plants were used for the experiments. Camelina pots were randomly arranged in the growth chamber, independent of their N treatment.

### 2.3 First trophic level—plants

Ten 30‐day‐old camelina plants per treatment were used for this experiment. The aerial part of each plant was cut and weighed using an electronic balance (Mettler Toledo ML204, Max: 220 g, *d* = 0.1 mg) (fresh weight). The first four leaves from the base of each plant were used to assess plant quality through the calculation of the Carbon:Nitrogen (C:N) ratio. Each plant sample was treated individually, lyophilized at 4°C for 48 hr (lyophilizer Alpha I‐5), grinded for 2 min with a ball mill (MM400, Restch, Germany), then placed in a tin basket and dry weighed (Sartorius Genius ME415S, Max: 410 g, *d* = 0.01 mg). The dosage of C and N was achieved using an elemental analyzer (Flash EA 1112 series Thermo Electron, Bremen Germany).

### 2.4 Second trophic level—aphids

#### 2.4.1 Nitrogen treatment and aphid performance

Pools of synchronized first‐instar nymphs (less than 24 hr old) of each aphid species were obtained from parthenogenetic adult females placed on leaves of *B. napus* set in 1.5% agar in Petri dishes (90 mm diameter). For the nymph survival study, groups of five‐first‐instar nymphs were transferred onto camelina plants. Each group of aphid nymphs was enclosed in a clip cage on one leaf at mid‐height of each camelina plantlet. Nymph survival was recorded daily. Twenty repetitions per aphid species × N treatment were made. For the adult performance study, for each treatment and each aphid species, 27–30 surviving adults were randomly selected and placed individually in a clip cage on the plant to be tested. Their survival and fecundity were recorded daily. The prereproductive period (i.e., the period of time from birth until the onset of reproduction) of adult aphids was also measured. Their fecundity was assessed for a duration equivalent to twice the prereproductive period duration (PRP) for each aphid species. Newly larviposited nymphs were removed daily (Hackett, Karley, & Bennett, 2013; Le Roux, Saguez, Vincent, & Giordanengo, 2004).

#### 2.4.2 Nitrogen treatment and aphid size

Thirty‐three to thirty‐five eight‐day‐old aphids, randomly selected from the nymph survival study, were measured under a stereomicroscope (LEICA M165C) from the tip of the head to the base of the cauda.

#### 2.4.3 Nitrogen treatment and aphid C:N ratio

Cohorts of eight‐day‐old aphids were obtained as described in the nymph survival study. For each aphid species, ten samples of 15–20 aphids per N treatment were frozen and then freeze dried for further C and N content analysis. The same protocol as the one used for leaf plant samples was used.

### 2.5 Third trophic level—parasitoid

The experimental setup used was modified from Boquel, Delayen, Couty, Giordanengo, and Ameline (2012). It consisted of six ventilated Plexiglas^®^ chambers (360 × 240 × 110 mm) used simultaneously, inside which, three plants of *C. sativa* (1 plant per pot) belonging to one of the three treatments (“0N”, “80N” or “240N”) were placed. In each chamber, 180–200 three‐day‐old nymphs, which had been reared on camelina from the corresponding treatment, were deposited evenly on the three plants. Groups of five to six males and 10 females standardized *D. rapae* wasps were introduced with a small paintbrush in the middle of the experimental setup for 24 hr before being removed. Two replicates of each treatment were performed. All experiments were conducted under controlled conditions (20 ± 1°C, 60% ± 5% RH, and 16L:8D photoperiod at 2.5 klux).

Aphids were observed daily until formation of a mummy. Mummies were transferred into plastic tubes (75 × 13 mm) closed with a cotton plug. Emerged parasitoids were sexed and then stored at −80°C for further measurements. Left hind tibia length was used as a proxy for parasitoid size. It was measured under a stereomicroscope (LEICA M165C) on 30–35 randomly selected parasitoid females for each N treatment × host aphid species combination.

The following parasitoid life‐history parameters were then computed: (1) nymphal developmental time (from mummification to adult emergence) in days; (2) emergence rate expressed as a percentage ((no. of emerged parasitoids/no. of mummies) × 100); and (3) tibia length (in mm) of parasitoid females.

### 2.6 Statistical analyses

Mean values are given with their standard error of the mean (*SEM*). The effect of treatment on plant (biomass and C:N) and aphid (PRP, fecundity and C:N) data that were not normally distributed was analyzed using a Kruskal–Wallis one‐way analysis of variance (*H*), followed by multiple comparison tests using the R package “nparcomp” (type: Tukey). Aphid survival was analyzed using a Cox‐proportional hazards model. Aphid length data being normally distributed, the effect of N treatment on this parameter was analyzed using a parametric one‐way ANOVA test followed by pairwise comparisons using permutational *t* tests (*p‐*value adjustment method: fdr, 999 permutations, package “RVAideMemoire”). The combined effects of aphid host species and N treatment on parasitoid parameters were analyzed using GLM with binomial error (link: logit) for emergence rate, as the use of binomial distribution is appropriate to model binary data or percentages; GLM with Gaussian distribution (link: identity) for the hind tibia length and Poisson distribution (link: log) for the development time. All statistical analyses were carried out using the statistical program “R” (R 3.2.2—R Development Core Team, 2015).

## 3 Results

### 3.1 First trophic level—plant

#### 3.1.1 Effect of nitrogen treatment on Plant aerial biomass and quality

**Figure 2:**
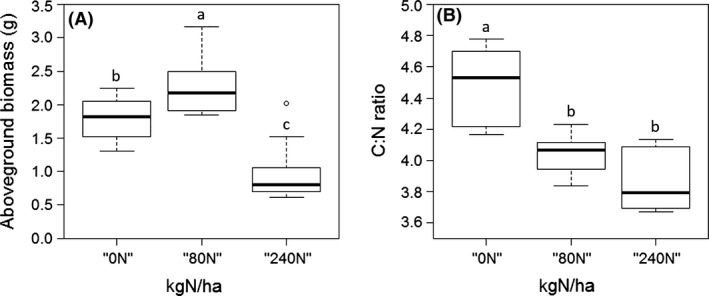
**(**A) Aboveground biomass and (B) Leaf C:N ratio of *Camelina sativa* plants exposed to different nitrogen treatments. The letters indicate significant differences associated with Kruskal–Wallis one‐way analysis of variance (*H*) followed by Tukey multiple comparison tests

There was a significant treatment effect on both the aerial biomass (Kruskal–Wallis test, *H* = 21.428, *df* = 2, *p *=* *2.223 × 10^−5^) and the C:N ratio (Kruskal–Wallis test, *H *=* *19.125, *df* = 2, *p* = 7.031 × 10^−5^). Compared to the control “0N” treatment, the aerial biomass of *Camelina sativa* plants was significantly higher under the “80N” treatment and significantly lower under the “240N” treatment (Fig. 2A). Both N treatments (“80N” and “240N”) induced a significantly lower foliar C:N ratio compared to the control (Fig. 2B).

### 3.2 Second trophic level—aphids

#### 3.2.1 Plant‐mediated nitrogen effects on aphid life‐history traits

**Figure 3:**
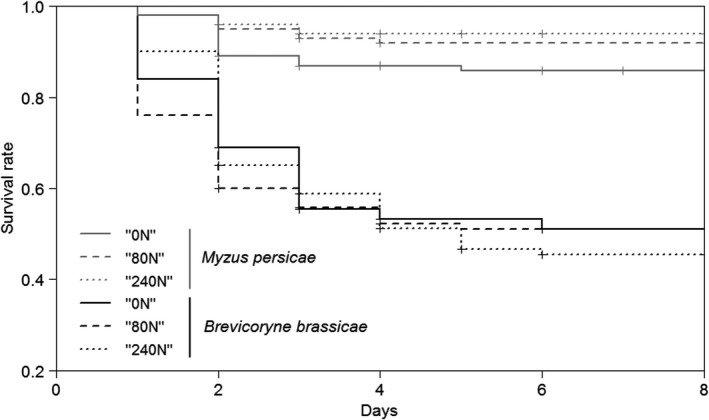
Survival rates of aphid nymphs of *Myzus persicae* and *Brevicoryne brassicae* on camelina plants exposed to different nitrogen treatments (“0N”, “80N,” and “240N”)

Whatever the treatment, nymph survival was significantly higher in *M. persicae* than in *B. brassicae* (ANOVA Cox model, *χ* ^2^ = 127.72, *df* = 1, *p *<* *2.2 × 10^−16^) (Fig. 3). There was no significant effect of the treatment on aphid nymph survival, neither for *B. brassicae* (ANOVA Cox model, *χ* ^2^ = 0.1501, *df* = 1, *p *=* *.698) nor for *M. persicae* (ANOVA Cox model, *χ* ^2^ = 3.438, *df* = 1, *p *=* *.064).

For *B. brassicae* (Table 1a), there was a significant effect of the treatment on both fecundity (Kruskal–Wallis test, *H* = 23.079, *df* = 2, *p = *9.7362 × 10^−6^) and length of adult aphids (ANOVA test*, F* = 6.380, *df* = 2, *p *=* *.006). The fecundity was significantly lower when aphids were reared on “240N”‐treated plants compared to “0N” control and “80N”‐treated plants. The length of *B. brassicae* adult aphids developed on N‐treated plants (“80N” or “240N) was significantly higher than on control plants. There was no significant difference between the PRP and the C:N ratio of adult aphids reared on the different plant treatments (Kruskal–Wallis tests, *H* = 0.841, *df* = 2, *p* = .657 and *H *=* *0.784, *df* = 2, *p *=* *.675).

**Table 1:** Life‐history traits (Mean ± *SEM*) of (a) *Brevicoryne brassicae* and (b) *Myzus persicae* aphids reared on *Camelina sativa* exposed to different nitrogen treatments (0, 80, and 240 kgN/ha)

Aphid parameters	“0N”	“80N”	“240N”	Statistics
(a) *Brevicoryne brassicae*				
*n*	29	30	30	
PRP (days)	9.172 ± 0.193a	9.300 ± 0.215a	9.633 ± 0.212a	*H* = 0.841, *df* = 2, *p *>* *.1
Fecundity (/)	50.793 ± 1.956a	44.533 ± 1.896a	33.333 ± 2.497b	*H* = 23.079, *df* = 2, ***p *** **<** *** *** **.001**
*n*	35	35	35	
Length (mm)	1.367 ± 0.021b	1.456 ± 0.017a	1.477 ± 0.027a	*F* = 6.380, *df* = 2, ***p *** **<** *** *** **.01**
*n*	10	10	10	
C:N ratio	5.72 ± 0.07a	5.86 ± 0.09a	5.87 ± 0.20a	*H* = 0.784, *df* = 2, *p *>* *.1
(b) *Myzus persicae*				
*n*	27	29	29	
PRP (days)	7.852 ± 0.163a	8.138 ± 0.136a	8.207 ± 0.235a	*H* = 2.087, *df* = 2, *p *>* *.1
Fecundity (/)	50.148 ± 3.346b	62.621 ± 1.781a	44.655 ± 2.316b	*H* = 23.996, *df* = 2, ***p *** **<** *** *** **.001**
*n*	34	33	33	
Length (mm)	1.393 ± 0.016b	1.448 ± 0.020a	1.362 ± 0.019b	*F* = 5.382, *df* = 2, ***p *** **<** *** *** **.05**
*n*	10	10	7	
C:N ratio	6.01 ± 0.10a	5.51 ± 0.10b	5.45 ± 0.13b	*H* = 9.715, *df* = 2, ***p *** **<** *** *** **.01**

*p*‐Values in bold indicate statistically significant differences with *H* (Kruskal–Wallis test) or *F* (ANOVA test). Kruskal–Wallis one‐way analyses of variance (*H*) were followed by Tukey multiple comparison tests and one‐way ANOVA tests were followed by pairwise comparisons using permutational *t* tests. The letters indicate significant differences associated with following pairwise comparisons. Means are presented with their standard error. *n*: number of aphids tested.

For *M. persicae* (Table 1b), there was a significant effect of the treatment on aphid fecundity (Kruskal–Wallis test, *H* = 23.996, *df* = 2, *p *=* *6.1572 × 10^−6^), the length of adult aphids (ANOVA test*, F* = 5.382, *df* = 2, *p* = .003), and the C:N ratio of adult aphids (Kruskal–Wallis test, *H* = 9.715, *df* = 2, *p* = .008). The fecundity was significantly higher when aphids were reared on “80N”‐treated plants compared to “0N” control and “240N”‐treated plants. The length of *M. persicae* adult aphids developed on “80N”‐treated plants was significantly higher than on control or “240N”‐treated plants. There was no significant difference between the PRP of adult aphids reared on the different plant treatments (Kruskal–Wallis test, *H* = 2.087, *df* = 2, *p* = .352). The adult aphids C:N ratio was significantly higher when aphids were reared on control plants (“0N”) than when they were reared on N‐treated plants (“80N” or “240N”).

### 3.3 Third trophic level—parasitoid

Emergence rate was significantly affected by the plant treatment (*χ* ^2^ = 22.695, *df* = 2, *p* = 1.1802 × 10^−5^). There was a significant interaction between aphid species and treatment (*χ* ^2^ = 22.047, *df* = 2, *p* = 1.6312 × 10^−5^). Only when *D. rapae* developed inside *M. persicae,* its emergence rate was significantly lower under the “0N” treatment (Table 2 a and b).

**Table 2:** Life‐history parameters (Mean ± *SEM*) of *Diaeretiella rapae* that developed inside (a) *Brevicoryne brassicae* and (b) *Myzus persicae* aphids on *Camelina sativa* exposed to different nitrogen treatments (0, 80, and 240 kgN/ha)

Parameters	“0N”	“80N”	“240N”
(a) *Brevicoryne brassicae*			
*n*	143	94	93
Emergence rate (%)	78.10	75.80	84.50
Development time (days)	14.25 ± 0.09	15.47 ± 0.15	14.33 ± 0.13
*n*	34	33	33
Hind tibia length (mm)	0.541 ± 0.005	0.555 ± 0.005	0.544 ± 0.006
(b) *Myzus persicae*			
*n*	70	101	73
Emergence rate (%)	55.60	88.60	85.90
Development time (days)	15.94 ± 0.16	15.79 ± 0.15	15.45 ± 0.14
*n*	32	31	31
Hind tibia length (mm)	0.547 ± 0.007	0.541 ± 0.007	0.572 ± 0.006

Means are presented with their standard error. *n*: number of parasitoids tested.

Development time was not significantly affected by the plant treatment but was affected by the aphid species in which *D. rapae* females developed, with reduced development time inside *B. brassicae* (*χ* ^2^ = 11.515, *df* = 573, *p* = .0007) (Table 2 a and b). There was no interaction between the two factors.

Hind tibia length was significantly affected by the N treatment (*χ* ^2^ = 0.007, *df* = 190, *p* = .038) but not by the aphid species (*χ* ^2^ = 0.002, *df* = 192, *p* = .1141). There was a significant interaction between aphid species and plant treatment (*χ* ^2^ = 0.015, *df* = 188, *p* = .0007). When *D. rapae* developed inside *M. persicae*, parasitoid hind tibia length increased only under the “240N” treatment while when *D. rapae* developed inside *B. brassicae*, there was no significant difference (Table 2a and b).

## 4 Discussion

Nitrogen inputs differently affected N concentrations within the first two trophic levels and the subsequent bottom‐up effects within our tritrophic system. Contrary to an optimal N application, when N was delivered in excess, the plant was stressed and its biomass decreased although N leaf concentration increased. Aphids parameters were negatively affected suggesting that the suitability of host plants receiving an excess of N was therefore reduced. Surprisingly, these negative effects resulted in no or positive effects on parasitoid parameters, suggesting a buffered effect at the third trophic level. Host N content, host suitability, and dietary specialization appear to be major factors explaining the functioning of our studied system.

### 4.1 Nitrogen host content and performances of superior trophic levels

Nitrogen input affected severely the biomass and physiology of camelina. In our study, plants receiving the optimal “80N” treatment had an increased aerial biomass and a decreased leaf C:N ratio, due to increased leaf N concentration (data not shown). Our results are in accordance with previous works (Hermans et al., 2006; Leith et al., 1999; Magill et al., 2000). For the excessive “240N” treatment, even if the C:N ratio decreased, plant biomass decreased indicating a stressed status of the plants. In some cases of excessive N input, ammonium (NH4+) stress may induce symptoms such as reduced growth or yield depressions (Britto & Kronzucker, 2002) and this type of stress may then cascade up negatively on the 2nd trophic level such as observed in our study.

Previously published papers have shown positive relationships between high N concentration in the host plant and high performances of phytophagous insects in terms of population growth rate (Couture, Servi, & Lindroth, 2010), weight increase (Winter & Rostás, 2010), or both (Aqueel & Leather, 2011). Consistent with these previous works, in our study, *M. persicae* size and fecundity and *B. brassicae* size were positively impacted by the higher leaf N concentration obtained in the optimal “80N” treatment. However, increased leaf N concentration obtained in the excessive “240N” treatment did not affect positively *M. persicae* size and fecundity nor *B. brassicae* fecundity. A study from Zehnder and Hunter (2009) examining the impact of experimental increases of foliar N levels has documented a diminution in performance of an aphid species, suggesting that excessive nutrient levels can limit herbivore population growth rates. This may occur when nutrient additions lead to host plants presenting nutrient ratios that exceed herbivore threshold elemental ratios (Boersma & Elser, 2006). To maintain homeostasis, herbivores need therefore to excrete excess elements, and if the excretion is energetically costly, then they may exhibit reduced population growth rates (Boersma & Elser, 2006). Other physiological mechanisms (reviewed in Zehnder & Hunter, 2009) such as a decrease of feeding rate, an alteration of assimilation efficiency, or the impact of plant composition on aphid symbionts, may also explain how excessive N concentrations may result in decreased aphid performances.

Fagan et al. (2002) have revealed an imbalance between herbivorous and parasitoid insect N concentrations, indicating that N limitation can influence higher trophic levels interactions (Denno & Fagan, 2003). On the one hand, on N‐treated plants N concentrations were enhanced for *M. persicae* but remained unchanged for *B. brassicae*. On the other hand, parasitoid emergence rates were enhanced when they developed inside *M. persicae* while emergence rates were not affected by the plant treatment when parasitoid developed inside *B. brassicae*. Therefore, in accordance with Aqueel et al. (2015), our study suggests that the greater is the N concentration in the body of the aphid host, the better is the parasitoid emergence rate. Effects of N input on development rate of parasitoid seem to be variable as both positive (Garratt, Leather, & Wright, 2010) and no effects (Aqueel et al., 2015) have been previously observed. In our study, whatever the N host content, the developmental duration of parasitoids was not affected. Interestingly, the size of female parasitoids was increased when developing in *M. persicae* in the excessive “240N” treatment, potentially enhancing the level of pest control they may provide as size is generally positively correlated to their fitness (e.g., egg load) (Jervis, Ellers, & Harvey, 2008).

### 4.2 Variation of N input and the “tritrophic interaction” hypothesis

According to the “tritrophic interactions” (TTI) hypothesis developed by Mooney et al. (2012): (1) generalist herbivores should be more sensitive to variation in host‐plant quality than specialist herbivores; and (2) a low‐quality host plant should impact more the third trophic level when the herbivore host/prey is a generalist than when it is a specialist. We therefore expected that (1) variations in performances would be more important for *M. persicae* than *B. brassicae*; and (2) the variation in host‐plant quality would affect more the parasitoid *D. rapae* developing on *M. persicae* than when it developed on *B. brassicae*.

#### 4.2.1 Validation of prediction (1) depends on the level of the N input

At the optimal “80N” treatment, consistent with prediction (1), the generalist *M. persicae* was more affected than the specialist *B. brassicae*. Fecundity and size of *M. persicae* were enhanced whereas in *B. brassicae* only the size was enhanced. However, unexpectedly, under the excessive “240N” treatment (i.e., stressed plant), prediction (1) was invalidated. *M. persicae* fecundity and size were not affected whereas, paradoxically, fecundity was reduced and, in the specialist aphid *B. brassicae*, size was enhanced. Both N treatments may have influenced host‐plant resistance to the second trophic level through the alteration of primary and secondary metabolites production (Chen, Schmelz, Wäckers, & Ruberson, 2008; Lou & Baldwin, 2004; Slansky 1992). For both N treatments, a decrease in the C:N ratio compared to the control treatment may be related to a higher percentage of amino acids in the phloem sap (Crafts‐Brandner, 2002). Extensive studies of aphid physiology have revealed the central role of sucrose concentration, amino acid concentration and composition, and sucrose:amino acid ratio in shaping aphid performances (Auclair, 1963; Douglas, 1998; Febvay et al., 1988). The better performance of *M. persicae* may therefore possibly be attributed to an increase in the quantity and/or quality of amino acids in the camelina plants submitted to the optimal “80N” treatment.

Several works have emphasized the role of bacterial symbionts in situation of low‐N availability. Indeed, aphids have bacterial symbionts that enable them to modify the quality of the ingested phloem sap by upgrading nonessential amino acids to essential amino acids (Douglas, 1998), enabling them to maintain high reproductive rates on diets with low concentrations of low‐quality nitrogen. However, there is no indication of how these microbial symbionts would behave in case of excessive N input and how this might alter their relationship with the aphids (Zehnder & Hunter, 2009).

Besides, high rates of N application may increase plant demand for water and then increase the possibility of water stress (Scagel, Bi, Fuchigami, & Regan, 2011). Many phytophagous insects, especially sap‐feeders, are adversely affected by continuous water stress (reviewed in Huberty & Denno, 2004). During periods of plant stress, reductions in turgor and water content may also interfere with the herbivore's ability to access or utilize nitrogen. This effect may also partly explain the limited fecundity of *B. brassicae* on the “240N” treatment.

Depending on the plant species, N treatment may also affect the synthesis and levels of constitutive and induced defensive secondary compounds (Chen et al., 2008; Dudt & Shure, 1994; Lou & Baldwin, 2004). For example, foliar glucosinolate content in Brassica crops is influenced by fertilizer treatments (Staley et al., 2010, 2011) and generally decreases in reaction to N input (Schonhof, Blankenburg, Müller, & Krumbein, 2007). The specialist aphid, *B. brassicae*, is dependent on glucosinolate compounds, which can act as phago‐stimulants (Cole, 1997a; Kim, Lee, Schroeder, & Jander, 2008) and may be or not affected by GLS in their host plants (Le Guigo, Qu, & Le Corff, 2011). N input may have induced lower glucosinolate concentrations in camelina leaves and then negatively affected *B. brassicae* feeding rate, thus leading to a decreased fecundity, whereas *M. persicae* performances, which are less strongly related to glucosinolate concentrations (Cole, 1997a), were enhanced. This latter result can be related to the way *M. persicae* deals with GLS. As a generalist aphid, it uses a strategy of toleration, whereby glucosinolates and other plant secondary metabolites are flushed rapidly through the aphid gut and are present in the honeydew (Weber, Oswald, & Zoellner, 1986).

Wilkens, Spoerke, and Stamp (1996) found that N fertilization altered levels of phenolic compounds that are associated with plant resistance to aphids (Cole, 1984). The decreased performance of *B. brassicae* on the “240N” treatment could therefore be attributed to a variation in such phenolic compounds.

#### 4.2.2 Validation of prediction (2)

Consistent with our prediction (2), whatever the N treatment, *D. rapae* performances were more affected, and positively so, when it developed on the generalist aphid *M. persicae* than when it developed on the specialist *B. brassicae*. It has been shown that plant allelochemicals may not only affect the development of herbivores but also that of their parasitoids (Hunter, 2003; Ode, 2006). Parasitoids of herbivores feeding on glucosinolate‐containing plants may be negatively affected by plant‐derived compounds that are stored in the hemolymph or other body tissues of the herbivore (Gols & Harvey, 2009). However, the lack of variation of the emergence rate of *D. rapae* developing in *B. brassicae* observed in our study can be related to the works of Le Guigo et al. (2011) which showed that *B. brassicae* did not benefit from the sequestration of GLS to limit attacks by *D. rapae*.

Nevertheless, performances improvement (i.e., emergence rate and size of the tibia) of *D. rapae* females when developing inside *M. persicae* could be explained by lower glucosinolates levels in camelina leaves exposed to N treatments. The better performances of *D. rapae* on *M. persicae* on N‐treated plants may be linked to the aphid host nutritional quality, expected to be enhanced on plant exposed to N treatment. Indeed, size of parasitoids is often related to nutritional characteristics of the host (Godfray, 1994) and adult life span and fecundity of the parasitoids are increased by the availability of nutrients in host insects (Azzouz, Giordanengo, Wäckers, & Kaiser, 2004; Kaneshiro & Johnson, 1996).

## 5 Conclusion

The originality of our work lies in the fact that although an excessive N input induced the expected negative impacts on the performance on the first two trophic levels, this effect was buffered at the third trophic level. This also highlights the fact that a high host N content does not always warranty a good nutritional quality for the upper trophic level.

Our work shows that according to the “tritrophic interactions” hypothesis, N treatments cascaded up differentially to the third trophic level depending on the aphid host dietary specialization. Variation in host‐plant quality had indeed stronger effects on dietary generalist aphids than on dietary specialist ones. Consequently, it appeared that bottom‐up forces were relatively more important on the parasitoids when a generalist aphid species was involved than when it was a specialist. This study provides key insight on the complex interactions between bottom‐up and top‐down forces that structure plant‐herbivores communities under the context of agricultural intensification. Indeed, N recommendations based on tests and projected crop N demand often result in over‐application (Cui, Zhang, Chen, Dou, & Li, 2010) and it seems that this excessive input, apart from being detrimental in terms of yield for the plant, could also have a significant impact on higher trophic levels.

## Funding Information

SAS PIVERT (Grant/Award Number: ANR‐001‐01).

## Conflict of Interest

None declared.
